# Development of camelid monoclonal nanobody against SLC39A6 zinc transporter protein

**DOI:** 10.22038/IJBMS.2021.58542.13003

**Published:** 2021-12

**Authors:** Hajarossadat Ghaderi, Zahra Noormohammadi, Mahdi Habibi-Anbouhi, Fatemeh Kazemi-Lomedasht, Mahdi Behdani

**Affiliations:** 1 Department of Biology, Science and Research Branch, Islamic Azad University, Tehran, Iran; 2 National Cell Bank of Iran, Pasteur Institute of Iran, Tehran, Iran; 3 Biotechnology Research Centre, Venom and Biotherapeutics Molecules Laboratory, Pasteur Institute of Iran, Tehran, Iran; 4 Zoonoses Research Centre, Pasteur Institute of Iran, Amol, Iran

**Keywords:** Breast cancer, Nanobody, Phage display, SLC39A6, VHH library

## Abstract

**Objective(s):**

SLC39A6 (solute carrier family 39) or LIV-1, is a zinc-transporter protein associated with estrogen-positive breast cancer and its metastatic spread. Significantly there is a direct relation between high zinc intake and unregulated cell proliferation and cancers. Blocking SLC39A6 protein may result in reduced metastasis and proliferation in many malignant tumors. This study aimed to develop an anti-SLC39A6 nanobody that is able to detect and block the SLC39A6 protein on the surface of cancerous cells.

**Materials and Methods:**

The recombinant SLC39A6 was expressed and used for camel immunization. The VHH library was constructed and screened for SLC39A6-specific nanobody. Then, the strength of nanobody in SLC39A6 detection was evaluated by Western blotting and flow cytometry.

**Results:**

We showed the ability of SLC39A6 specific Nanobody (C3) to detect SLC39A6 by Western blotting and flow cytometry. Furthermore, the C3 nanobody potently inhibits cell proliferation in MTT assay.

**Conclusion:**

These data show the potential of SLC39A6-specific nanobody for the blockade of zinc transporter protein and provide a basis for the development of novel cancer therapeutics.

## Introduction

Given the changing pattern of life and increasing the risk factors, cancer is currently the second leading cause of death in developed countries and the third leading cause of death in developing countries such as Iran. Breast cancer is the most common cancer among women in Iran and the world, and its incidence is expected to increase dramatically in the coming decades (1). Most treatment costs are spent on the three breast, lung, and prostate cancer types, with costs increasing as the disease progresses to its highest stage (2). 

There are three main methods for treating breast cancer: tissue resection surgery, chemotherapy, and radiotherapy, which have some side effects. Attention to the high costs, long duration of treatment, the side effects of common treatments of breast cancers, and necessity of research into new diagnostic and therapeutic approaches are clear (2). During two past decades, a lot of studies for the treatment of breast cancer was done; these studies have shown that the expression of SLC39A6 increased in ER (estrogen receptor) positive breast cancer patients (3). SLC39A6 is one of the most important members of the ZIP (zinc transporter) family. The zinc transporter family, in mammalians, consists of two groups, the zinc transporter (ZnT) or solute carrier 30 (SLC30) family and the zinc importer, Zrt- and Irt-like protein (ZIP), or solute carrier 39A (SLC39A) family. ZnT protein reduces zinc concentration in the cytoplasm, and ZIP protein increases zinc content in the cytoplasm (4). In physiological conditions, the zinc transporter is abundantly expressed in organs such as the breast, prostate, placenta, kidney, pituitary, and corpus callosum (5, 6). SLC39A6 protein transports zinc into the cytosol via the plasma membrane, contains a histidine-rich transcriptional domain that binds to zinc, and aids in its transport into the cell (5). SLC39A6 is up-regulated in pathological hormone-rich tissues, including breast and uterine cancer, where it is thought to be influenced by cellular inducement and may play an important role in tumor development and metastasis (7). In recent years, it has been made clear that down-regulation of SLC39A6 significantly inhibits cell proliferation and reduces tumor growth (8). Given that increased expression of SLC39A6 is implicated in lymph node involvement in breast cancer, it is suggested that it may play a role in metastasis (9). Recent studies have determined SLC39A6 can be a good candidate for both tumor markers and antibody-drug conjugate (ADC) treatment (10). 

Due to the side effects and high costs of common cancer therapy and the non-specificity of this treatment, new methods such as immunotherapy by antibody have been used today. Among the different types of antibodies, the kind that known as Nanobody is one of the most interesting for researchers. There is a specific type of antibody in the camel’s serum, (heavy-chain antibody or HcAb), which consists of only two heavy chains and does not have a light chain. Nanobody or VHH comes from the variable domains of HcAb (11). The physical and pharmacological properties of nanobodies such as low immunogenicity for humans, high stability, good aquatic solubility, and small size which enable easy handling for genetic engineering and expression in microorganisms have led to their application in cancer diagnosis and treatment (12, 13). 

In this study, we tried to find a cheaper and easier way to treat cancers which is directly linked to estrogenic hormones by preparing specific nanobody against the SLC39A6 protein.

## Materials and Methods

***Preparation of recombinant SLC39A6***

The SLC39A6 extracellular domain gene was provided in pET22b by Bagheri *et al* (14). The construct was transformed into *Escherichia coli *BL-21, induced with 0.1 mM Isopropyl β-D-1-thiogalactopyranoside (IPTG), and purified by Immobilized Metal Affinity Chromatography (IMAC) using Ni^2+^ ions immobilized on the resin (Ni-NTA) method. The protein purity was assessed by SDS-PAGE and Western blotting (by anti-His tag antibody). 

***Camel immunization***

A 7-month young female camel was immunized by six-time injection of 100 μg of purified recombinant SLC39A6 protein with a two-week interval. In the first injection, complete Freund’s, and for booster injection, incomplete Freund’s adjuvants were used. For assessment of camel immune response, blood was collected before each injection and the serum was kept in a -20 °C freezer. ELISA and Western blotting by using recombinant SLC39A6 were done with this serum.

***VHH Library construction***

Five days after the last immunization, about 250 ml camel blood was collected and subsequently used to extract white blood cells by the ficoll separation method. RNA was extracted by RNeasy mini kit (QIAGEN, Germany) and cDNA synthesized according to Accut power cycle script RT remix (Bioneer, Korea). Nested PCR was performed by two sets of primers; Call001 and Call002 for primary PCR and A6E and 38 for nested amplification (15). VHH genes were cloned in pHEN4 phagemid by *Not*I and *Pst*I enzymes. The ligated product was transformed into *E. coli TG1* by electroporation. Transformant were cultured on Luria-Bertani (LB) agar supplemented with 100 μg/ml ampicillin and 2% glucose. The bacterial library was harvested and glycerol stock was stored at -70 °C. The efficiency of library construction was controlled by serial dilution of transformant and colony-PCR (15). 

***Phage display and bio-panning***

The bacterial library was cultured in 2xYT (2× yeast extract tryptone) broth medium with 2% glucose and 100 μg/ml ampicillin and infected by 10^12 ^VCSM13 helper phage, subsequently incubated overnight at 37 °C. The recombinant phage (rPhage) was precipitated by PEG-NaCl solution (20% PEG6000, 2.5M NaCl). Three cycles of bio-panning were conducted in the solid phase. Ten micrograms of recombinant SLC39A6 protein were coated in 96 well ELISA plates and blocked with 4% skim milk. 10^11 ^rPhage was added to the well and incubated at RT (room temperature) for 1 hr. The ELISA wells were washed 10, 20, and 25 times with PBST (Phosphate Buffer Saline with 0.05% tween 20) in the first, second, and third rounds of panning, respectively. The specific phage that was attached to coated antigen was eluted with 100 µl of triethylamine solution (pH 10) and neutralized with 1M Tris solution. Eluted rPhage was used for reinfection and qualitative assessment of enrichment process. 

***Qualitative and quantitative assessment of the enrichment process***

After each round of panning 20 µl of eluted rPhage was diluted serially and used for *E. coli* TG1 infection. The colony counted in each dilution and enrichment ratio was calculated and compared with the uncoated well. For quantitative assessment, 10^10 ^rPhage from each round were used for ELISA with SLC39A6 antigen. The well was coated and blocked, then phages were added. Anti-M13 Horseradish Peroxidase (HRP) conjugated was used as antibody and the color was developed with TMB (3, 3′, 5, 5′-Tetramethylbenzidine). 

***Monoclonal VHH selection***

Ninety-six single colonies were cultured in 1 ml LB Broth and induced with 0.1 M IPTG. The periplasmic protein (PE) was extracted by osmotic shock (16) and used for ELISA. Each ELISA well was coated by recombinant SLC39A6 protein, and PE was applied. Anti-HA and anti-mouse HRP conjugated were used as primary and secondary antibodies, respectively. The colonies that had minimum two-fold increases in comparison with negative (uncoated) wells were chosen and their plasmid was DNA sequenced. 

***SLC39A6 specific nanobody expression***

The selected VHH gene entitled C3 was sub-cloned to pHEN6C plasmid. The single colony was cultured in terrific broth ( TB) with 100 µg/ml ampicillin, 0.2% glucose, and 0.2M MgCl_2_ in final concentration. The culture was induced with 0.1M IPTG. The periplasmic proteins were extracted by osmotic shock and applied on Ni-NTA resin. The purified nanobody was confirmed with ELISA and Western blot. 

***Affinity measuring***

For Affinity measuring, Beatty’s method (17) was used. Two concentrations of antigen (SLC39A6 protein) 1 μg/well and 10 μg/well were coated in ELISA plate and blocked with skim milk 4% for 1 hr in RT. Serial dilutions of nanobodies (10pM to 10nM) were added and incubated at RT for 1 hr. Rabbit anti-camel antibody (1:2000) and goat anti-rabbit HRP conjugated (1:2000) were added subsequently. TMB was added and OD 450nm was read. The k_aff_ affinity of nanobody was measured by using Beatty’s formula:

[Ag]/[Ag^,^] = N

k_aff_ = (N –1)/2(N[Nb] – [Nb^,^])

***SLC39A6-specific nanobody specificity***

The ELISA method was used for determination of nanobody specificity. Seven different antigens such as human programmed cell death protein 1 (hPD1), mouse programmed cell death protein 1 (mPD1), human programmed death-ligand 1 (hPDL1), mouse programmed death-ligand 1 (mPDL1), human cytotoxic T-lymphocyte-associated protein 4 (hCTLA4), mouse cytotoxic T-lymphocyte-associated protein 4 (mCTLA4), B-cell activator factor (BAFF), and SLC39A6 in 0.5 μg/well concentration were coated. ELISA was developed by adding SLC39A6 specific-nanobody, rabbit anti-camel antibody, and goat anti-rabbit HRP conjugated and TMB.

***Cell lysate Western blotting***

10^7 ^cells of MCF-7 and HEK293 were lysed by heat shock and were loaded on 12% SDS-PAGE. The cell lysate protein was transferred to nitrocellulose and blocked. Five micrograms of SLC39A6 specific-nanobody were added to the paper and incubated overnight at room temperature. Then rabbit anti-His (1:2000) and goat anti-Rabbit HRP conjugated (1:1000) were used respectively. The color was developed by 4-Choloro1-naphthol.

***Flow cytometry***

SLC39A6-expressing cells (MCF-7), are used for flow cytometry along with a cell line that is negative for SLC39A6 expression (Jurkat). Test was done in three microtubes that are prepared for each cell line, one for colorless cells (negative control), one for commercial antibody (positive control) and one for SLC39A6-specific Nanobody. In each microtube, 10^6 ^cells were added and washed with PBS. Two micrograms of commercial antibody or C3 nanobody were added to the corresponding tubes and incubated at 4 °C for 30 min. Then rabbit anti-His and goat anti-rabbit fluorescein isothiocyanate (FITC) conjugated antibodies were used. After washing and resuspension, all tubes were analyzed by a Partec cyflow flow cytometer. 

***MTT assay***

About 2×10^4^ MCF7 cells per well were cultured and incubated at 37 °C with 5% CO_2 _overnight. Cells were exposed to C3 Nb (100, 70, 35, 17, 8, 4 and 2 µg). After 72 hr, MTT solution (3-[4, 5-dimethylthiazol-2-yl]-2, 5-diphenyltetrazolium bromide) was added to the wells and incubated at 37 °C for 4 hr. Then the MTT solution was removed from the wells and 100 μl of dimethyl sulfoxide was added and incubated at 37 °C for 30 min. The optical density was read at 570 nm. The assay was performed in triplicate and the percentage of toxicity of C3 nanobody was calculated based on the following formula: (%) =1- [100 × (sample abs)/ (control abs)].

## Results

***Recombinant SLC39A6 expression***

**Figure 1:**
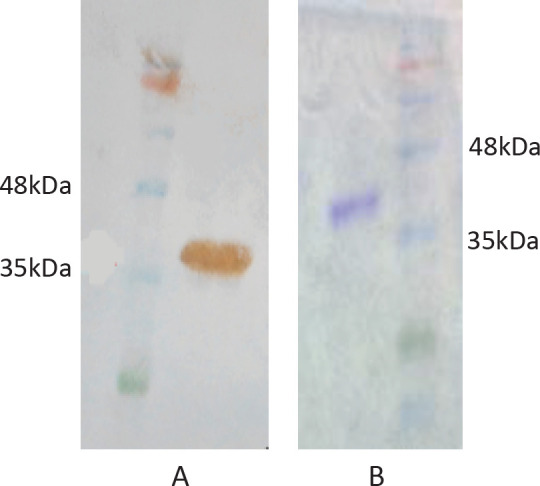
SDS-PAGE (A) and Western blotting (B) of purified recombinant SLC39A6 protein

The extracellular domain of SLC39A6 was cloned and expressed in the bacterial expression system; the recombinant protein was expressed and purified. SDS-PAGE and Western blotting were done and the high-purity protein that is positioned at 36 kDa was confirmed (Figure 1).

***Phage library construction and bio-panning***

**Figure 2:**
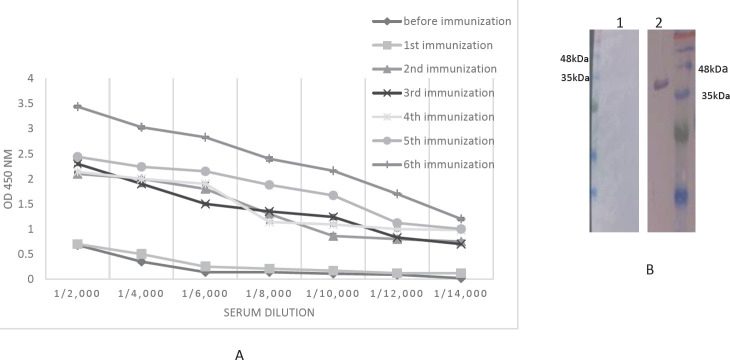
Serum ELISA (A) and Western blotting (B) for camel immunization process monitoring. Western blotting was done with camel serum before immunization (lane 1) and after fifth injection (lane 2)

One female *Camelus dromedarius *was immunized with recombinant SLC39A6 six times with a two-weeks interval. The immunization process was followed by ELISA and Western blotting. The result confirmed an increase in the level of serum antibodies after the second and 6^th^ immunizations (Figure 2). 

**Figure 3:**
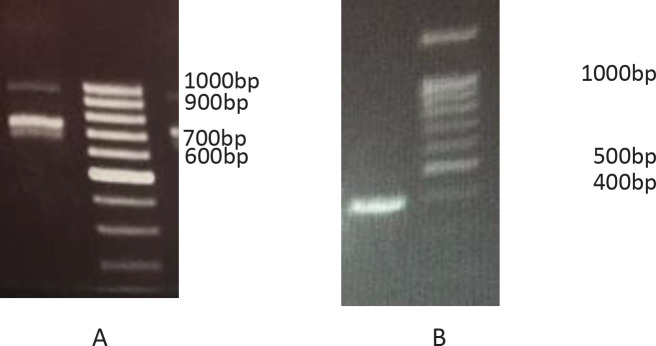
VHH gene amplification by nested PCR. First PCR by specific primers for VHH leader sequence and CH2 domain (A). Nested PCR with framework-1 and framework-4 specific primers (B)

**Figure 4:**
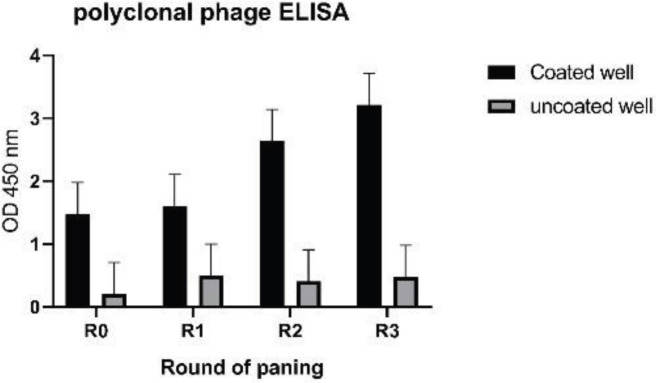
Qualitative assessment of panning procedure by polyclonal phage ELISA. R0; Phage library, R1; round 1 output phage, R2; round 2 output phage, R3; round 3 output phage

For preparing the VHH library, RNA was extracted from peripheral blood lymphocytes and cDNA was synthesized. The first PCR shows two bands for conventional antibody (900bp) and heavy chain antibody (600–700bp) (Figure 3A) and in nested PCR, the 400bp band of nanobody was obtained (Figure 3B). The nanobody gene was cloned in pHEN4 phagemid and transformed to *E. coli *TG1. The size of bacterial library by colony counting method measured and it was 7.5 ×107 CFU. The efficacy of cloning was measured by colony-PCR and was about 91.4% Three rounds of panning was done, and its process was evaluated by quantitative and qualitative methods. Colony counting in serial dilution of eluted phage from antigen coated and uncoated wells, was used for qualitative method (Table 1). The enrichment factor of third round was 1030 and more than 100 fold first round. The result of polyclonal phage ELISA (quantitative method) that showed in 3th round, the specific phage is highest (Figure 4).

**Figure 5:**
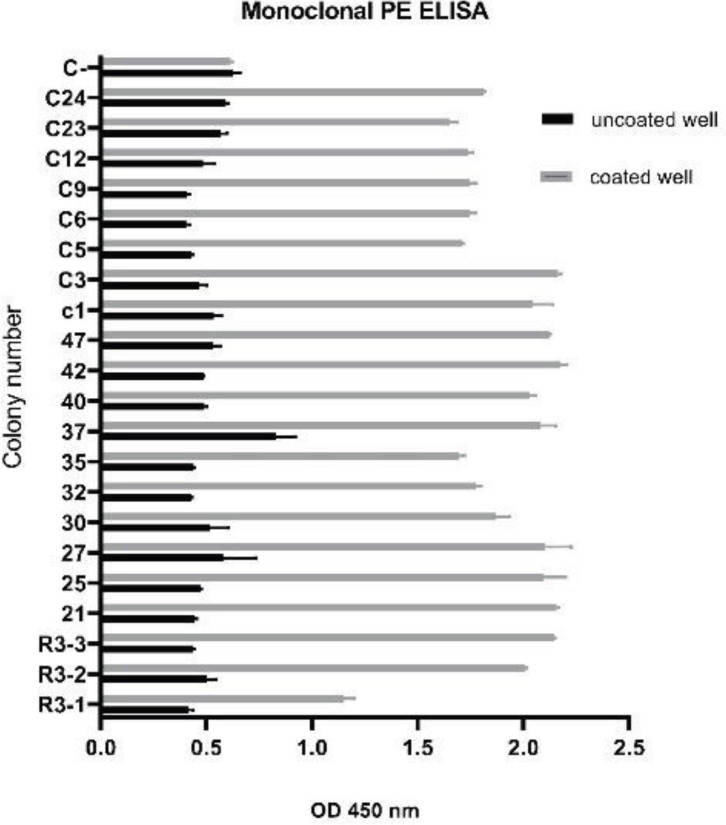
Monoclonal PE-ELISA. Twenty-three colonies had more than 2-fold differentiation between uncoated and coated wells

**Figure 6:**
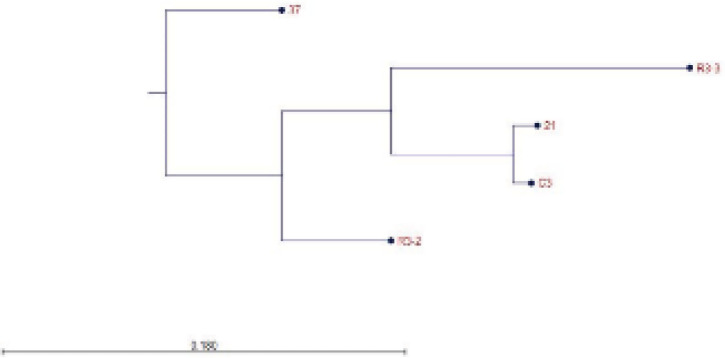
Phylogenetic tree by CLC software showed that the 37 nanobody comes from different origins but R3-3, 21, C3, and R3-2 were from the same origin

**Figure 7:**
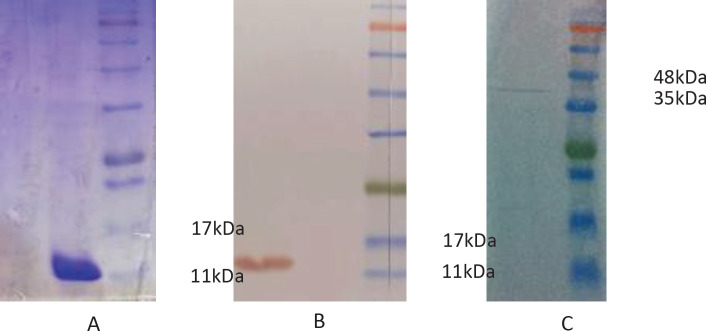
Western blotting (A) and SDS-PAGE (B) of purified nanobody, and SLC39A6 protein detection by C3 nanobody on Western blotting (C)

From 96 colonies which selected by PE-ELISA, 23 colonies had more than 2-fold differentiation between uncoated and coated wells (Figure 5). Based on the repeatable result and considering on the differentiation between negative and positive wells the six colony R3-2, R3-3, C3, C21, C26, and C37, with highest OD was selected and DNA sequencing was done. Finally, the selected nanobody sequence was classified by CLC software (Figure 6) and the C3 clone was selected for further test. The gene of C3 Nb was sub-cloned to pHEN6 plasmid and transformed to *E. coli *Wk6. The expression of recombinant protein was done by IPTG and recombinant nanobody was purified by Ni-NTA affinity chromatography. The SDS-PAGE and Western blotting confirmed the nanobody with about 15 kDa (Figures 7A, B). The final expression yield was about 15 mg/L. Purified C3 nanobody could determine the SLC39A6 in Western blotting (Figure 7C).

***Nanobody characterization***

*Specificity*

**Figure 8:**
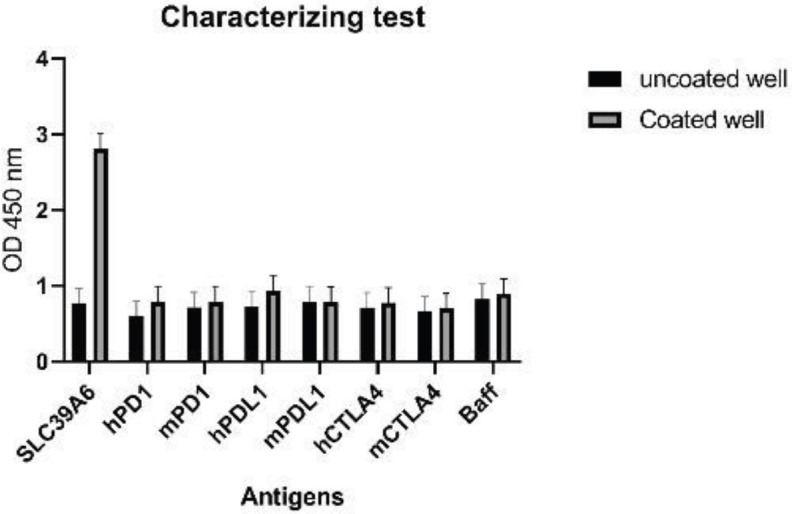
ELISA for the specificity of C3 nanobody on seven different antigens

The specificity of C3 Nb was evaluated in ELISA whereby the binding to the SLC39A6 was compared to seven other proteins such as mPD-1, hPD-1, mPDL-1, hPDL-1, mCTLA-4, hCTLA-4, Baff (Figure 8). A low background signal was observed on all non-SLC39A6 proteins. Whereas C3 Nb exhibited a very strong signal with SLC39A6 that reached close to 4-fold the background.

*Affinity measurement*

**Figure 9:**
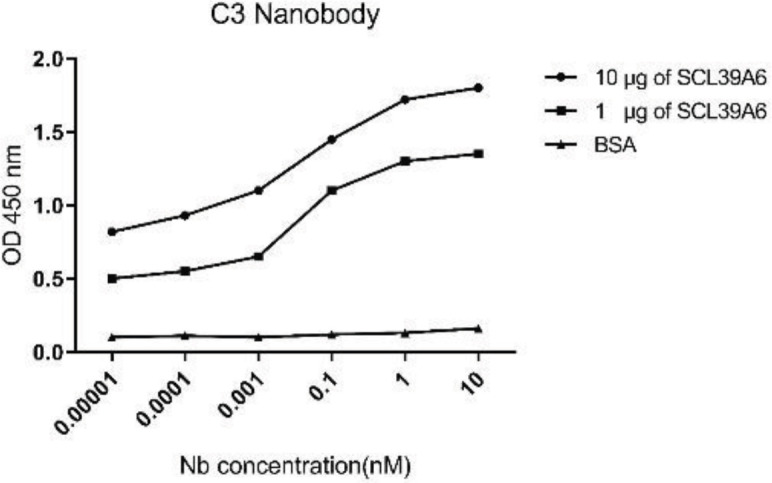
Binding affinity of the C3 nanobody to the SLC39A6 protein

The affinity of the selected nanobody towards the SLC39A6 antigen was estimated by ELISA, using two different amounts of antigen immobilized in the wells. The Nbs were added to the wells at concentrations ranging between 10pM to 10nM. The ELISA test was done in triplicate. The *K*_aff_ of nanobody was obtained from Beatty’s methods. Based on the formula, the affinity constant was 1.7 ×10^4^ nM (Figure 9).

***Evaluation of SLC39A6-binding potential***

*Using of C3 Nb for Western blotting*

**Figure 10:**
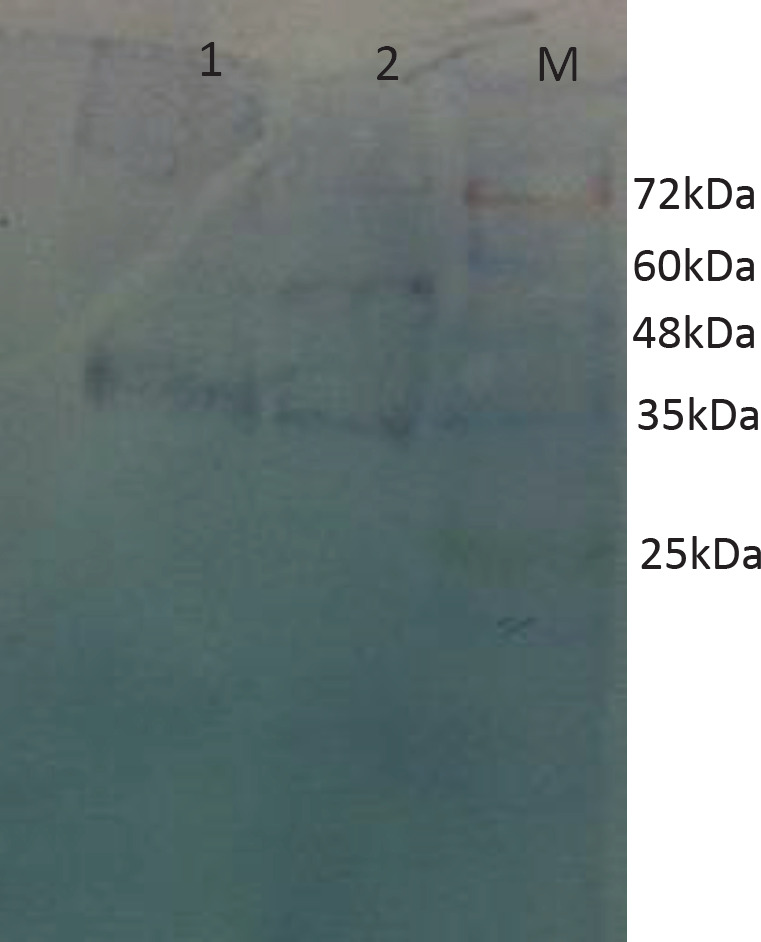
Detection of SLC39A6 with C3 Nb on cell lysate by Western blotting. 1; MCF-7 cell lysate, 2; HEK293 cell lysate, M; protein marker

Western blotting was used to investigate the potential of C3 nanobody to detect the SLC39A6 surface protein on a solid membrane. The MCF7 and HEK293 cell lysate were run on SDS-PAGE and transferred to nitrocellulose paper. The C3 nanobody was used as the first antibody and anti-camel and anti-rabbit HRP as second and third antibody. It was showed C3 Nb could detect SLC39A6 protein in western blotting (Figure 10).

*Analyzing the attachment of C3 Nb to SLC39A6 on the cell surface*

**Figure 11:**
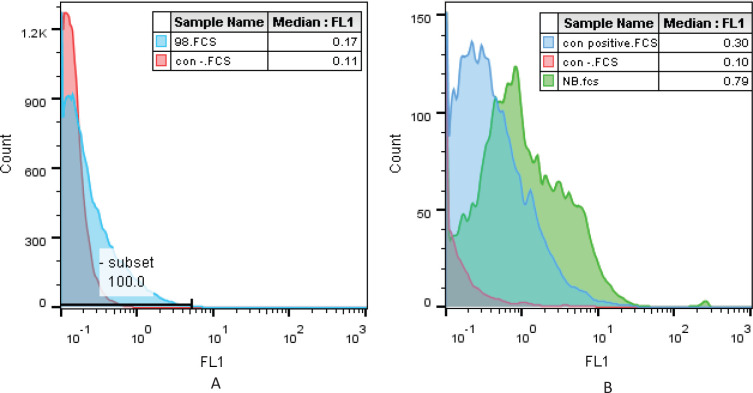
C3 Nb cell-binding assessment on Jurkat cell as a negative cell (A) and MCF-7 cell as a positive cell (B). Blue; C3 Nb in (A) and commercial anti-SLC39A6 in (B), Red; cell without Nb, Green; C3 Nb in (B)

Comparison of the C3 Nb binding to the native cell surface antigen by flow cytometry indicated that the obtained C3 Nb from the library binds better than the commercial antibody. Nanobody results showed 86% and commercial antibodies 80% binding to the antigen. In contrast, no binding was observed in the Jurkat cell line due to lack of expression of SLC39A6 on its surface (Figure 11).

***Bioassay***

**Figure 12:**
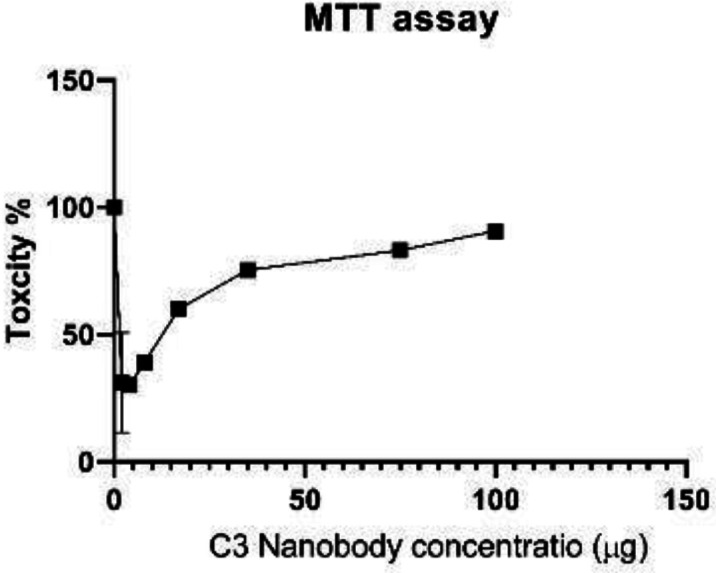
Effects of C3 nanobody on MCF-7 proliferation

MTT assay was used for showing the toxicity of C3 Nb on cancerous cell. In this study, the MCF-7 cell line was used. According to the results of the test, the increasing concentration of C3 Nb caused decreasing viability and increasing toxicity (Figure 12). Based on the graph of MTT, IC_50_ of nanobody on MCF-7 was measured at 12 μg. 

**Table 1:** Qualitative assessment and the ratio of colony numbers in coated well in comparison with uncoated well from the bio-panning procedure

**Enrichment rate **	**Uncoated well**		**Coated well**		**Round of panning**
	**No. of colonies**	**Dilution factor**	**No. of colonies**	**Dilution factor**	
**3.5**	9	-1	32	-1	**R1**
**20**	1	-4	2	-5	**R2**
**1030**	1	-3	103	-4	**R3**

## Discussion

Cancer statistics show that the number of cases of breast cancer in women and prostate cancer in men has dramatically increased, and it is mainly related to developing countries (18). There are three main ways of breast cancer therapy. Surgery and tissue removal are the common methods, which can lead to malformations and deformities of the breast. There is also the possibility of recurrence after surgery, therefore, requiring complementary therapies. The second method, radiotherapy, as a complementary therapy can cause chest pain, respiratory problems, dry swelling coughs, and fibrosis of the Chemotherapy also uses drugs that can cause heart problems, increased incidence of other cancers, gastrointestinal problems, hair loss, and severe pain. In addition, hormone therapy is currently being studied, but the use of hormones reduces bone density, bone weakness, and even bone loss, which is a cause for concern (19). SLC39A6 is the zinc transporter protein that overexpresses in estrogen-positive breast cancer and causes increasing zinc concentration in the cytoplasm. Some studies showed that increase of zinc transporter on the surface of the cell is involved with metastases of cancer and involving lymph nodes (20). It seems the SLC39A6 could be a good target as a diagnostic of metastatic breast cancer during the disease (21). These characterize SLC39A6 which make it a good target for immunotherapy methods. Recently, Ladiratuzumab vedotin, an antibody-drug conjugate (ADC) that targets SLC39A6, is in phase II trial that disrupts cell microtubulin and cause’s apoptosis (22). 

Monoclonal antibodies (mAbs) are a promising class of antibodies that can overcome the limitations of other therapeutics. mAbs have several advantages, such as a long-circulating half-life, utilized as drug carriers, targeting a toxic payload to cancer cells with an extremely high affinity for their targets, which is eligible with respect to pharmacodynamics. mAbs has shown great prospects in improving the antitumor effect in immunotherapy which is nowadays FDA approved at about 100 mAbs.

In 1997, a new type of antibody called nanobody was discovered; nanobody comes from a camelid antibody that does not have a light chain, and it has only a heavy chain. The physicochemical properties of nanobodies have made them useful tools for biomedical applications and drug discovery. It is due to their small size that this feature also offers nanobody as a tool that can efficiently cross the blood-brain barrier (23), also a high binding affinity for secretive epitopes, low immunogenicity, and cost-effective production (24). There are currently several nanobodies in different phases of clinical trials, such as Vobarilizumab, a monovalent nanobody against IL6 receptor in phase II trials for rheumatoid arthritis and systemic lupus erythematous. Also, different nanobodies were developed against tumor molecules; some of them target the growth factor receptors such as the epidermal growth factor receptor 1, 2 (EGFR-1, 2) (25), vascular endothelial growth factor receptor-2 (VEGFR-2)(26), human epidermal growth factor receptor-2 (HER-2) (27), mesenchymal-epithelial transition factor (c-Met) (28) and chemokine receptor (29), and others target the growth factors such as hepatocyte growth factor (HGF) (30) and vascular endothelial growth factor (VEGF) (31).

Since the discovery of the phage display technique, this technology has been regarded as a strong tool in investigation. The advantages of this technique include safety, ease of operation, simplifying of the cloning steps, low cost, phage stability, storage at 4 °C for a long time, high speed, and making various libraries like single pot and immune libraries. Eventually, by screening systems like bio-panning screening, particular antibodies can be selected without ethical concerns.

In contrast to other types of mAb libraries, for the preparation of nanobodies, we employed phage display technology to obtain SLC39A6-specific nanobodies from a camel-derived immune nanobody library.

One of the most common methods to prepare monoclonal nanobody is screening of immune phage libraries (32). Immune libraries are constructed from immunized Camelidae against a particular target. An advantage of this kind of library is that the v-genes contain matured affinity and the disadvantage of the development of this type of library can be restricted to ethical constraints and smaller size of the library than universals library (33). 

The selected nanobody was isolated from three continuous rounds of bio-panning, in each round for gaining more specificity, incubation time and washing times increased. Furthermore, the obtained enrichment factor was about 1000, which is a valid amount compared with other similar immune nanobody library studies (34). A noticeably increasing rate of OD in polyclonal phage ELISA can approve the efficiency of the bio-panning trend. 

In this study, nanobody against SLC39A6 protein was selected to be used as diagnostic and therapeutic tool in breast cancer which was prepared from an immune library. The C3 Nanobody was capable of detecting SLC39A6 at the surface of cells, it possesses that can be used as a marker of SLC39A6 breast cancer. 

The range of nanobody affinity is 10^− 2^ to 10^− 4^ for *the* *k*_off_ rate (35). Based on the results of the affinity test C3 nanobody has The C3 Nanobody was capable of detecting SLC39A6 at the surface of cells, it possesses that can be used as a marker of SLC39A6 breast cancer. to SLC39A6. Furthermore, C3 Nb could be distinguished and characterized SLC39A6 from the other antigen that was shown in the specificity test. On the other hand, the results of the MTT assay show the toxicity of this nanobody for breast cancer cell. To the best of our knowledge, this is the first report on SLC39A6 that by using the immune displayed phage library we can success isolated Nanobody.

## Conclusion

C3 Nb that was developed in this study has hallmark characteristics of an antitumor agent in terms of proliferation inhibition. The identified Nb could be helpful for revealing the role and function of SLC39A6 in malignancy conditions and to evolve towards a new candidate for clinical applications. Moreover, computational methods could be used for rational selection of the best Nbs as an efficient, cost and time benefit approach. 

## Authors’ Contributions

HG, MB Study conception and design; ZN, FK Data analyzing and draft manuscript preparation; MB, MH Critical revision of the paper; ZN, MB Supervision of the research.

## Conflicts of Interest

The authors declare that no conflict of interest exists.
